# Supplementary material for: Temporal and spatial heterogeneity of host response to SARS-CoV-2 pulmonary infection
Source: Nat Commun. 2020 Dec 9;11:6319. doi: 10.1038/s41467-020-20139-7 (PMC7725958; doi:10.1038/s41467-020-20139-7)
Supplement: Supplementary file 1 — Supplementary Information [file 41467_2020_20139_MOESM1_ESM.pdf]

LIST OF INVESTIGATORS

Niyati Desai[1,*], Azfar Neyaz[1,*], Annamaria Szabolcs[1,*], Angela R. Shih[2,*], Jonathan H. Chen[1,2], Vishal Thapar[1], Linda T. Nieman[1], Alexander Solovyov[5], Arnav Mehta[1,4], David J. Lieb [4], Anupriya S. Kulkarni[1], Christopher Jaicks[1], Katherine H. Xu[1], Michael J. Raabe[1], Christopher J. Pinto[1], Dejan Juric [1], Ivan Chebib [2], Robert B. Colvin[2], Arthur Y. Kim[3], Robert Monroe[6], Sarah E Warren[7], Patrick Danaher[7], Jason W Reeves[7], Jingjing Gong[7], Erroll H Rueckert[7], Benjamin D. Greenbaum[5], Nir Hacohen[1,3,4], Stephen M. Lagana[8], Miguel N. Rivera[1,2,4], Lynette M. Sholl[9], James R. Stone[2,#], David T. Ting[1,3,#], Vikram Deshpande[1,2,#]

[1] Massachusetts General Hospital Cancer Center, Departments of [2]Pathology and [3]Medicine, Boston, MA 02114, USA
[4] The Broad Institute, Cambridge, MA 02142, USA
[5] Memorial Sloan Kettering Cancer Center, New York, NY 10065, USA
[6] Advanced Cell Diagnostics, a Bio-Techne Brand, Newark, CA 94560, USA
[7] NanoString Inc., Seattle, WA 98109, USA
[8] Columbia University Irving Medical Center, Department of Pathology and Cell Biology, New York, NY 10032, USA
[9] Brigham and Woman's Hospital, Department of Pathology, Boston, MA 02115

* Equal contribution

Table of Contents

Figures S1 – S6
Tables S1-S11
Supplementary Data 1 Summary qRT PCR, RNA- ISH and RNA-Seq data
Supplementary Data 2 Genes in each cluster for Figure 2
Supplementary Data 3 Differential gene expression of high vs low virus cases

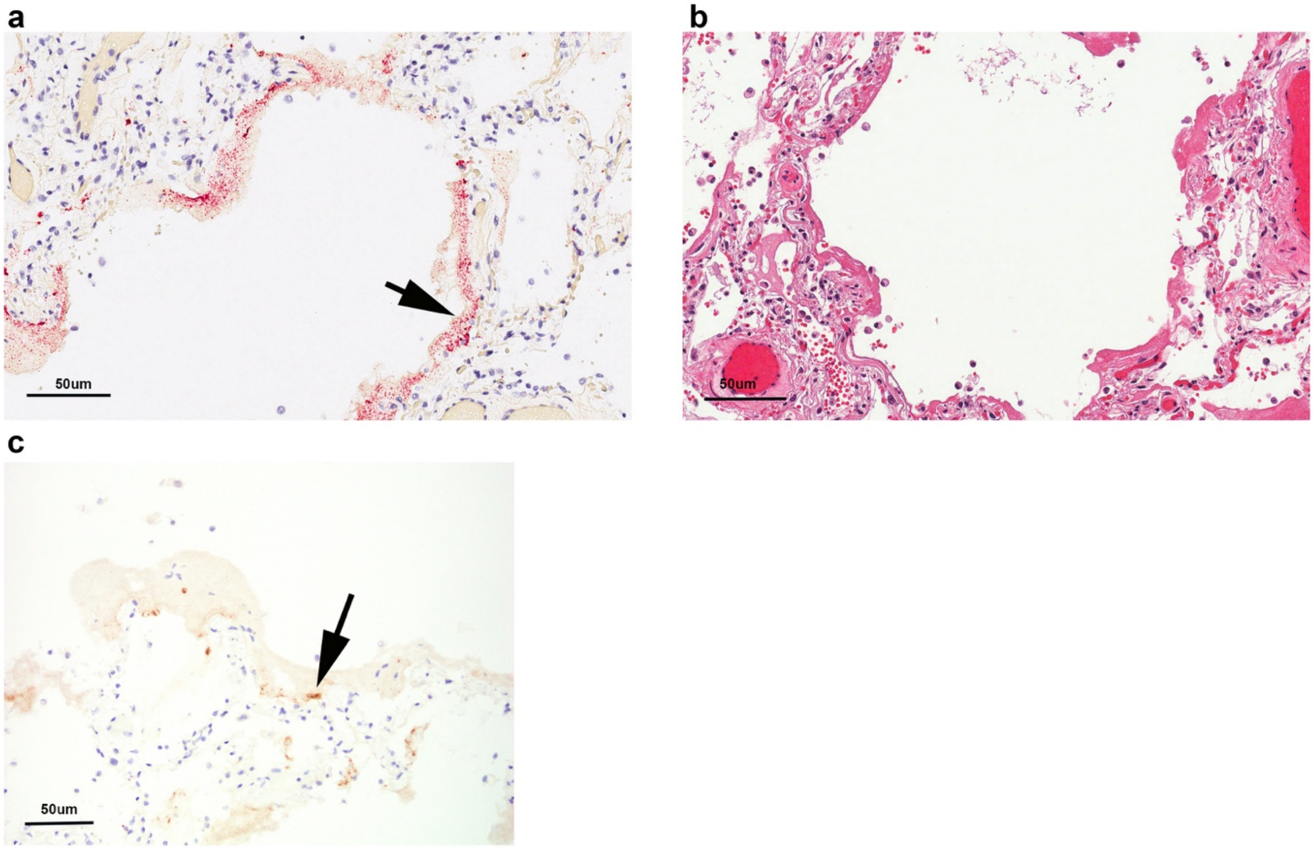

Supplementary Figure 1

a Extracellular viral RNA (arrow) in hyaline membranes (RNA-ISH for SARS-CoV-2).**b** H&E stain of Viral RNA High case. **c** Immunohistochemical stain for SARS-CoV-2. The antibody cross reacts with SARS-CoV-2. Note that the pattern of staining (arrow) is similar to that seen in Panel a.

all images are 40X, scale bar = 200um.

a

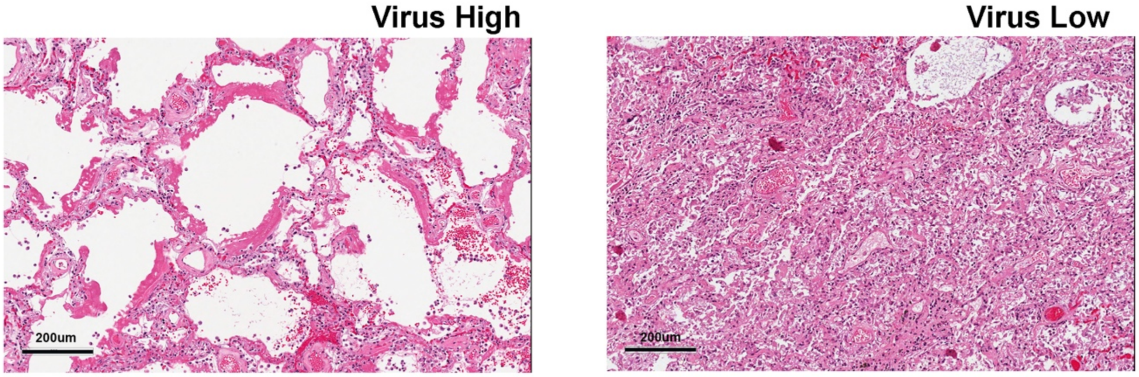

b

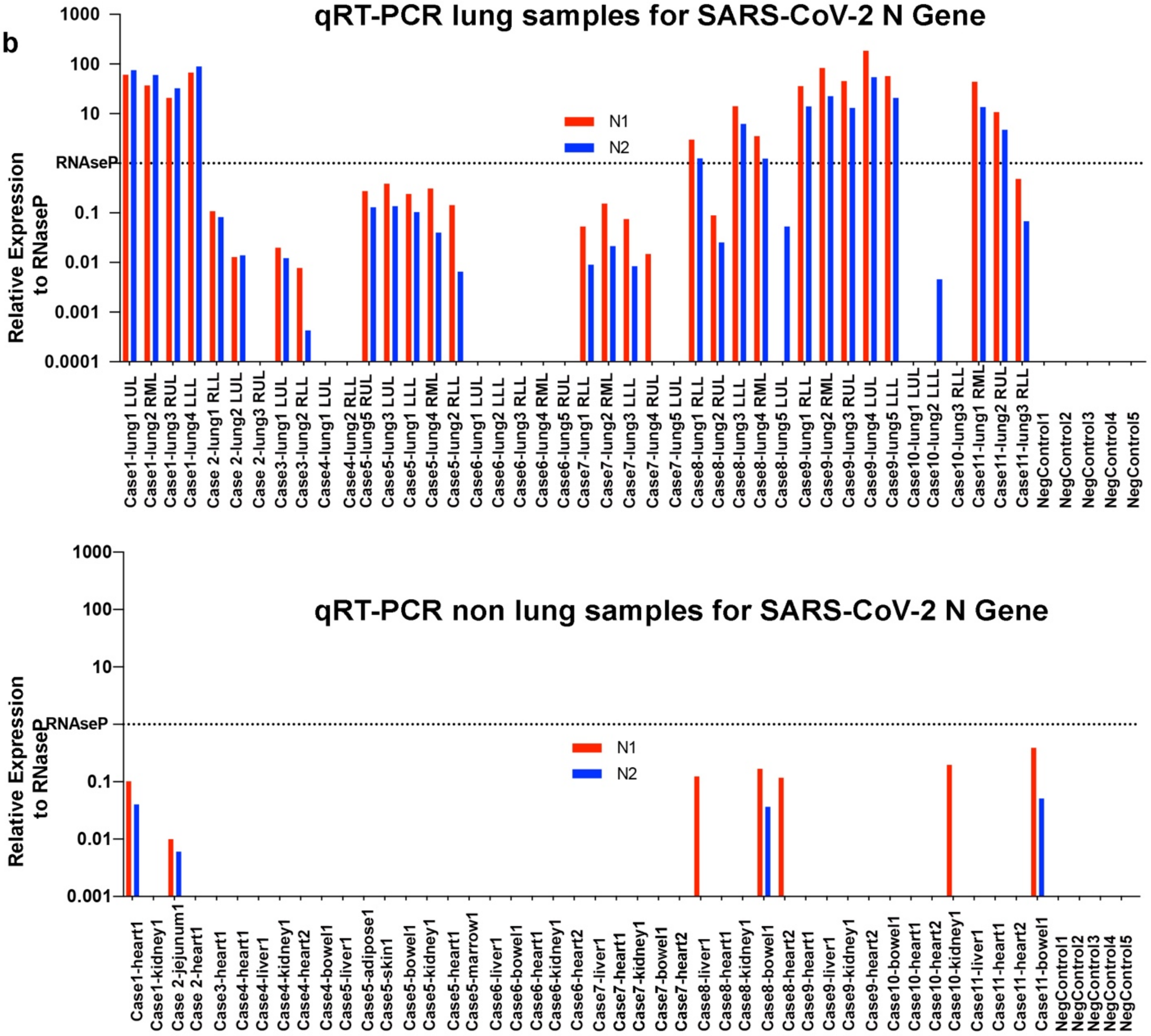

Supplementary Figure 2

a Representative H&E of SARS-CoV-2 Viral RNA High and Low cases. Image 10X, scale bar = 200um.
b qRT-PCR for SARS-CoV-2 N gene expression relative to RNaseP from autopsy cases. Source data are provided as a Source Data file

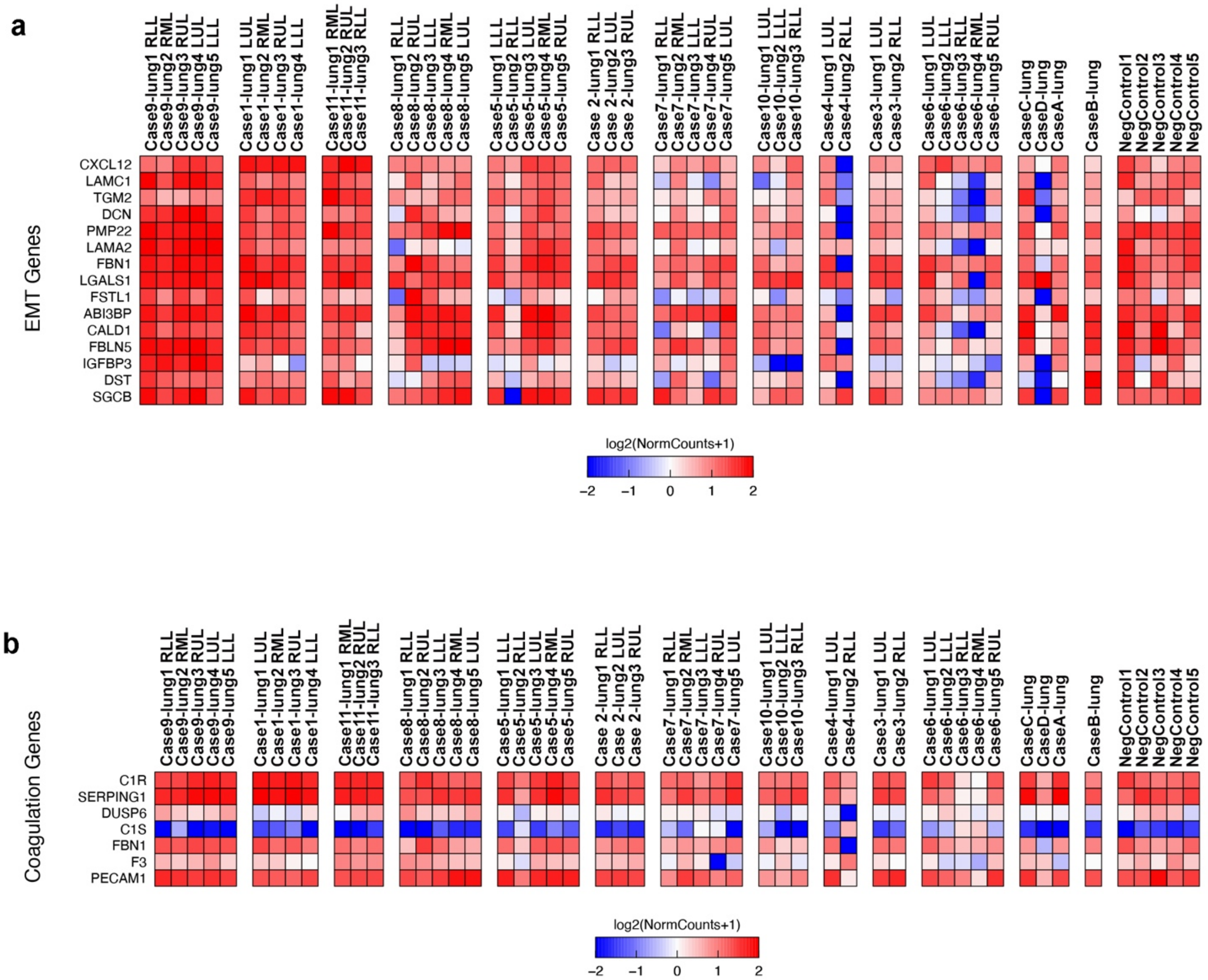

Supplementary Figure 3

Expression heatmap of genes significantly higher in high viral cases and enriched for genes in **a)** EMT and **b)** coagulation from the Hallmark gene set from MSigDB. Genes shown were statistically significant with FDR < 0.01. Source data are provided as a Source Data file.

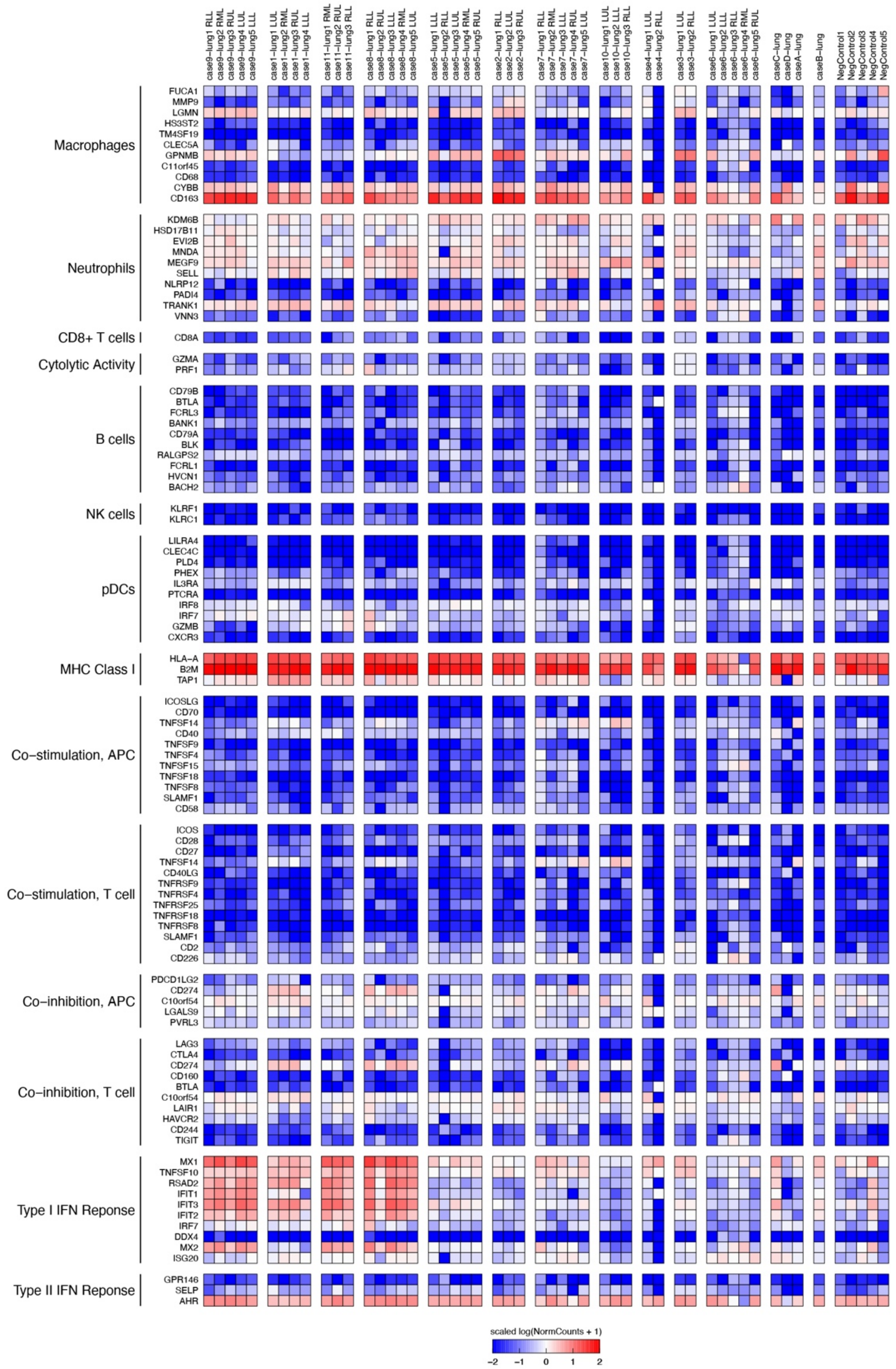

Supplementary Figure 4

Expression heatmap of immune genes in SARS-CoV-2 Viral RNA High and Low cases and controls. Source data are provided as a Source Data file.

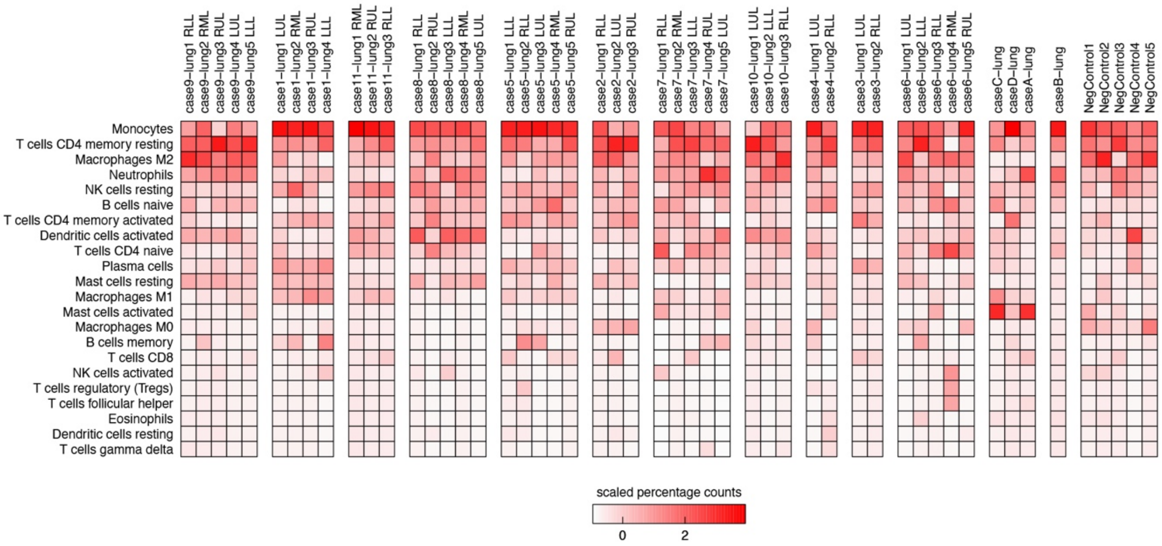

Supplementary Figure 5
Estimated percent of each cell type using RNA-seq deconvolution (CIBERSORTx) in each sample. Cell type ordered by highest to lowest average percent composition. Source data are provided as a Source Data file.

a

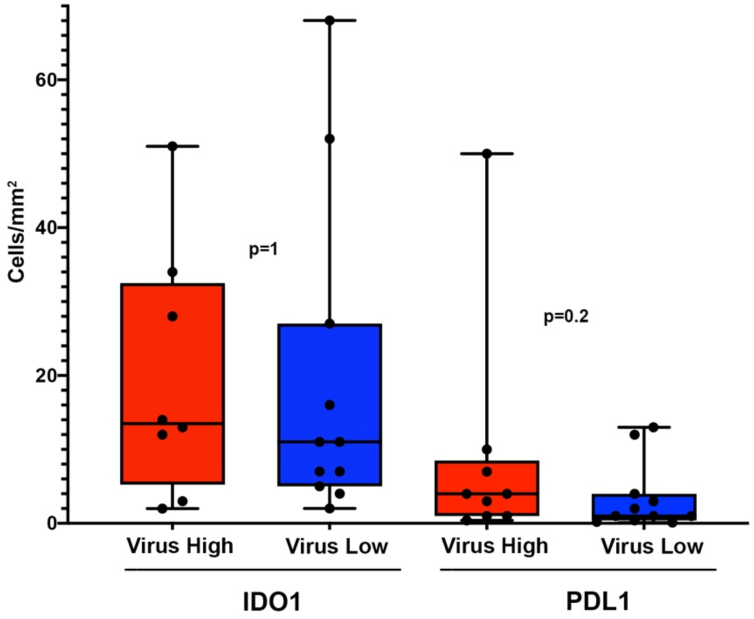

b

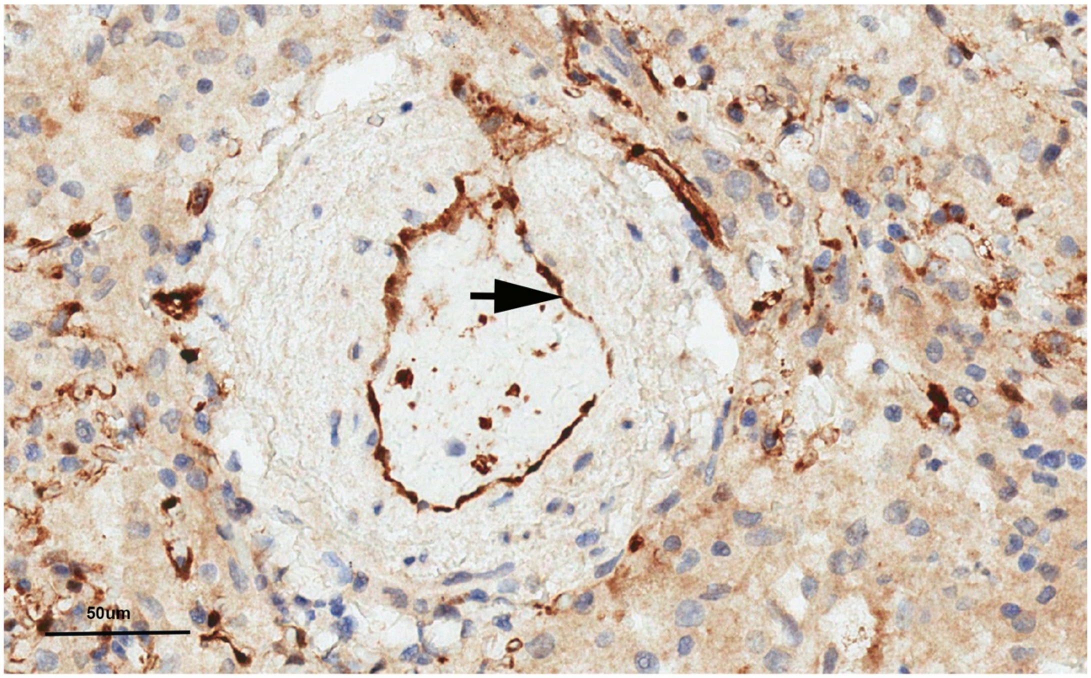

Supplementary Figure 6

a Box-plot graph of IDO1 and PDL1 IHC quantification. (one section per case. n=20). Box-and-whisker plot, center line, median; box limits, upper and lower quartiles; whiskers, range. P-value two tailed t-test.
b IDO1 IHC showing prominent endothelial staining(arrow). Image 40X, scale bar = 50um. Source data are provided as a Source Data file.

Supplementary Table 1. Clinical and demographic data

Case No.	Age (decade) & Gender	Presenting symptoms & Signs	Pre-existing diseases	Medication on admission	Immune suppression	COVID19 and related treatment	Duration of illness (days)	Time from Admission to Death (days)	Mechanical ventilation
Case 1	70s/F	Fever, lethargy, hypoxemic	T2DM, HTN, Psoriasis, Obesity, Osteoarthritis	Methotrexate, Atorvastatin, Insulin, Citalopram, Losartan	Yes, on methotrexate	Hydroxychloroquine Ceftriaxone Azithromycin	7	5	No
Case 2	70s/M	Fever, cough, myalgia, sore throat, hypoxemic	Asthma, Metastatic bladder cancer, Prostate cancer	Allopurinol, Colchicine, Coumadin, Imipramine	No	Hydroxychloroquine Atorvastatin Vancomycin Cefepime Metronidazole	18	13	Yes
Case 3	50s/M	Fever, cough, hypoxemic	T2DM, NAFLD, Hypothyroidism Obesity, Sciatica	Atorvastatin, Metformin, Dulaglutide, Fenofibrate, Hydrochlorothiazide, Losartan, Levothyroxine, Prednisone	Prednisone	Hydroxychloroquine Atorvastatin Ceftriaxone Azithromycin	13	12	Yes
Case 4	60s/F	Fever, cough, headache, myalgias, hypoxemic	T2DM, HTN, Hypothyroidism, Latent TB	Metformin, Levothyroxine, Gabapentin, Latanoprost, Duloxetine	No	Hydroxychloroquine Atorvastatin Azithromycin	20	6	Yes
Case 5	60s/M	Fever, fatigue, cough, dyspnea	T2DM, HTN	allopurinol, amlodipine, aspirin, atorvastatin, cholecalciferol, empagliflozin, lisinopril, metformin, sildenafil	No	Hydroxychloroquine Atorvastatin Ceftriaxone Azithromycin	12	5	Yes
Case 6	50s/M	Fever, cough, myalgia, nausea, loose BM, hypoxemic	Renal transplant in 2016, T2DM, HTN, HLD, CAD, CHF	Tacrolimus, Mycophenolate mofetil, Prochlorperazine, Pravastatin, Labetalol, Insulin, Prednisolone	Yes	Hydroxychloroquine Ceftriaxone, Azithromycin, Pravastatin	23	16	Yes
Case 7	70s/F	Unresponsive, Lethargy, tachycardia, hypotension, hypoxemic	Alzheimer's disease, T2DM, HTN, HLD, GERD	Memantine, Pravastatin, trazodone, escitalopram	No	cefepime , vancomycin	NA	1	Yes
Case 8	30s/M	Lethargic, Fever, rhinorrhea, weakness	Developmental delay, Autism, Epilepsy, T2DM, Bipolar	Trazodone, Benztropine, Clonidine, Haloperidol, Quetiapine, Melatonin	No	Hydroxychloroquine Azithromycin	6	3	Yes/mask ventilation

			affective disorder, Psoriasis						
Case 9	70s/F	Weakness, nausea, diarrhea, abdominal pain, hypotension	Alzheimer's disease, CKD, Hypothyroid, HTN, GERD, treated metastatic ovarian cancer, prolong QT, Panhypopituitarism	Amlodipine, citalopram, donepezil, labetalol, melatonin, levothyroid, prednisolone, polyethylene glycol	Yes, prednisolone	Cefepime	9	7	No
Case 10	30s/F	Fever with chill, cough, hypoxemia, homeless	HTN, OSA, Obese	Lisinopril, hydrochlorothiazide	No	Hydroxychloroquine Atorvastatin Ceftriaxone Azithromycin	9	7	Yes
Case 11	70s/M	NA	Lewybody Dementia, HTN, CKD, Prior CVA, Addison's Disease, GERD, Sarcoidosis	Amantadine, Atorvastatin, Chlorpromazine, Clonazepam, Prednisolone, Gabapentin, Melatonin, Quetiapine	Yes, prednisolone	NA	NA	NA	No
Case 12	40s/M	Fever with chills, Profound hypoxemia	T2DM, HCL, GERD	Metformin, Insulin, Atorvastatin,	No	Atorvastatin Ceftriaxone Azithromycin, Plaquenil	20	7	Yes
Case 13	40s/M	Fever, sore throat, cough, lossofsmell, myalgia, SOB	None	None	No	Hydroxychloroquine Ceftriaxone Azithromycin,	21	15	Yes
Case 14	80s/M	Weakness, confusion, delirium fever, fatigue	Dementia, HTN, Asthma, UC,	Amlodipine, salmeterol, Montelukast, Sulfasalazine, Simvastatin, Theophiline	No	Hydroxychloroquine Azithromycin, Atorvastatin	18	15	No
Case 15	80s/F	Lethargic, confusion, Sore throat, SOB, hypoxemia	H/O of colon cancer, CLL, T2DM, HTN, HLD,	Aripiprazole, Bupropion, Diltiazem, Sertraline, Trazodone	No	Simvastatin, Cefepime, Vancomycin	7	5	No

			Achalasia						
Case 16	40s/F	NA	Pulmonary sarcoidosis, Neuropathy, HTN, GERD, Extreme obesity with alveolar hypoventilation, steroid induced DM	acetaminophen, citalopram, amitriptyline, carvedilol, Insulin, oxycodone, pregabalin, meclizine	NA	NA	NA	NA	No
Case 17	80s/M	Cough, Fever, Syncope, Nausea	HTN, COPD, BPH, Sleep apnea	Atenolol, Lisinopril, Lovastatin	No	Hydroxychloroquine, Atorvastatin	17	14	Yes
Case 18	40s/F	Confusion, cough, vomiting,	Fibromyalgia, breast cancer, CNS Vascular malformation	Gabapentin	No	Acyclovir Propofol	2	1	Yes
Case 19	50s/M	Fever, cough myalgia, hypoxemia	HLD, Obesity, Asthma,	loratadine, polyethylene glycol	No	Hydroxychloroquine Azithromycin, Ceftriaxone Atorvastatin	27	17	Yes
Case 20	70s/M	Fever, Cough, rhinorrhea, SOB,	Dementia,	Sertraline, Quetiapine,	No	Hydroxychloroquine	23	10	Yes
Case A	70s/M	Essentially dead on arrival in ER. No History available	NA	NA	NA	NA	NA	0	NA
Case B	30s/F	ulcer/ osteomyelitis	T2DM, chronic DVT, osteomyelitis	Insulin	NA	NA	NA	32	NA

Case C	80s/M	SOB	CAD, HTN, COPD, prostate carcinoma	Albuterol, Amlodipine, Aspirin, Atorvastatin, Desmopressin, Gabapentin, Meclizine, Isosorbide Mononitrate, Metoprolol, Mylanta, Oxybutynin, Oxycodone, Acetaminophen Tamsulosin	No	No	NA	1	NA
Case D	50s/F	NA<br>BIBA intubated	HTN, HLD, DM	Albuterol Sulfate, amlodipine, Aspirin, Atorvastatin, Desmopressin, Gabapenti, Tylenol	No	No	NA	2	yes

Abbreviations: Neg= negative, NA= not available, SOB = shortness of breath, AKI= acute kidney injury, CAD= coronary artery disease, COPD= chronic obstructive pulmonary disease, HLD= Hyperlipidemia, CHF= congestive heart failure, GERD= gastrointestinal reflex disease, OSA= obstructive sleep apnea, HCL= hairy cell leukemia, UC= ulcerative colitis, CLL= chronic lymphocytic leukemia, BPH= benign prostatic hypertrophy, DVT = deep vein thrombosis, T2DM= type 2 diabetes mellites, HTN= hypertension, NAFLD= non-alcoholic fatty liver disease BIBA=brought in by ambulance

Supplementary Table 2. Lab parameters

Case No.	TG (mg/dl)	LDH (U/L)	AST (U/L)	CPK (U/L)	D dimer (ng/ml)	FG (mg/dl)	aPTT, PT (sec)	PC (ng/ml)	Ferritin (ug/L)	CRP (N<8)	IL6 (pg/ml)	CBC (WBC,Hb,Pt)	WBC Diff (P, L, M)	ALC (K/ul)
Case 1	NA	442	42	388	3592	NA	NA	6.8	675	313	NA	8,12.8, 168	P91L8M1	0.6
Case 2	242	521	121	188	3688	1241	>150, 18.5	3.68	758	238	103.9	19.1, 11.3, 520	P74L5M13	1
Case 3	676	477	229	1124	3625	1300	80.4, 16.5	30.03	1177	403.5	>3000	57.4, 9.9, 257	P74L8M5	4.8
Case 4	369	4719	1906	94073	>10000	469	57.8, 21.9	NA	25000	260.7	1204.9	16.5,11.8, 33	P89L4M2	0.6
Case 5	356	1111	1553	14879	3652	807	72.3, 15.1	7.6	5182	148.2	>400	20.2, 11.4, 25	P79L8M3	1.6
Case 6	857	662	206	4008	3677	651	40,16	0.11	7325	303	305	3.4, 16.5, 71	P83L11M7	0.5
Case 7	NA	519	72	309	>10000	NA	NA	2.7	4398	70	NA	16.1, 5.0, 218	P87L4M4	0.6
Case 8	NA	302	55	1539	2198	641	40.5, 15.8	6.9	798	299	NA	19.0, 3.9, 128	P81L7M10	1.4
Case 9	163	684	57	367	4323	536	37.4, 14.6	9.2	511	131	21.5	19.4, 12.3, 67	P93L4M2	0.8
Case 10	1252	581	49	771	22022	710	100.4, 15.7	0.3	413	13	61.8	30.9, 11.4, 213	P75L11M4	3.4
Case 11	NA	NA	NA	NA	NA	NA	NA	NA	NA	NA	NA	NA	NA	NA
Case 12	380	374	85	42	4909	761	36.4, 17.8	NA	2768	145.2	NA	9.9, 11.8,463	P59L27M12	2.7
Case 13	498	567	120	2103	1786	1000	63.4, 16.9	0.1	3961	124.4	44.4	39.2,11.4,249	P86L9M2	3.4
Case 14	89	438	26	784	3789	855	-, 16.6	0.1	351	251	7.7	7.7, 11.3, 243	P74L17M7	1.3
Case 15	NA	580	NA	1209	1349	NA	NA	0.1	1064	139.3	NA	24.4,14.2,399	P41L55M2	13.5
Case 16	NA	NA	NA	NA	NA	NA	NA	NA	NA	NA	NA	NA	NA	NA

Case 17	121	313	58	161	1784	627	14.2, 84.9	0.1	464	135.3	NA	17.2, 11.1, 240	P82L7M5	1.2
Case 18	NA	519	42	282	2608	733	14.8, 31.7	0.9	690	68.7	NA	30.2, 21.3,319	P87L2M6	0.6
Case 19	274	511	159	3121	4159	654	-,30.4	0.2	615	141.6	NA	16.8, 8.2, 309	P80L9M7	1.5
Case 20	139	300	184	92	1783	995	NA	15	NA	204	NA	11.2, 8.9, 284	P77L8M10	0.9
Case A	NA	NA	NA	NA	NA	NA	NA	NA	NA	NA	NA	NA	NA	NA
Case B	193	687	12	NA	7	496	15,23	NA	706	158	NA	9.8, 6.8, 271	NA	NA
Case C	NA	NA	146	NA	NA	NA	NA	NA	NA	NA	NA	NA	NA	NA
Case D	NA	853	120	NA	9.87	NA	36,16	15.48	773	>300	NA	NA	NA	NA

Triglycerides=TG, Low density lipoprotein=LDL, Aspartate aminotransferase=AST, Creatine Phosphokinase=CPK, Fibrinogen=FG, Procalcitonin=PC, Absolute lymphocyte counts=ALC, Procalcitonin=PC, Absolute lymphocyte count=ALC

Supplementary Table 3. SOFA and qSOFA score

Case	Intubated/on Ventilator	Virus High low	Admitted in ICU	SOFA score	qSOFA
1	No	High	No	1	0
2	yes	Low	Yes	11	1
3	yes	Low	yes	13	2
4	yes	Low	yes	11	2
5	yes	High	yes	10	2
6	yes	Low	yes	12	1
7	yes	High	yes	NA	1
8	yes/mask ventilator	High	yes	13	1
9	No	High	yes	NA	3
10	yes	Low	yes	13	2
11	No	High	NA	NA	NA
12	yes	Low	yes	11	2
13	yes	Low	yes	9	3
14	No	Low	No	NA	0
15	No	High	No	NA	1
16	No	High	No	NA	NA
17	yes	Low	yes	13	1
18	yes	High	yes	6	1
19	yes	Low	yes	10	1
20	yes	Low	yes	13	1

* For all intubated patients, Verbal GCS 1 considered.

Supplementary Table 4. Quantitative RNA-ISH and IHC analysis on lung samples

Case No	Viral high vs. viral low*	Viral load (% positive tissue area by RNA-ISH)	ISH hyaline membrane reactivity	RNA seq coverage	qRT-PCR**	Keratin cells / mm2	Napsin A cells / mm2	CD1 63 cells/ mm2	CD 3 Cells/ mm2	CD 4 cells/ mm2	CD8 cells/ mm2	CD20 cells/ mm2	CD123 cells/ mm2	CD18 cells/ mm2	CD56 cells/ mm2	IDO1 cells/ mm2	PD-L1 cells/ mm2
1	High	81.2	Present	99.96	Positive	192	174	216	174	98	24	17	6	3	59	51	50
2	Low	0.5	Absent	9.40	Positive	335	429	1026	420	225	78	21	63	17	31	68	13
3	Low	2.0	Absent	1.27	Positive	663	474	855	956	354	212	51	14	3	25	52	1
4	Low	<0.01	Absent	0.15	Negative	1045	680	723	290	152	39	23	31	7	54	27	1
5	High	18.5	Present	24.27	Positive	478	183	1010	386	203	65	12	4	2	20	28	10
6	Low	0.02	Absent	0.17	Negative	198	294	317	167	50	10	15	3	0.4	24	11	0.2
7	High	6.2	Present	10.45	Positive	273	265	546	395	135	139	21	22	2	56	34	4
8	High	23.5	Present	45.16	Positive	241	208	51	113	69	44	24	14	2	104	14	4
9	High	89.7	Present	99.98	Positive	1045	139	570	201	100	95	13	15	3	22	13	7
10	Low	0	Absent	1.52	Inconclusive	233	72	421	144	51	15	5	8	3	9	5	0.1
11	High	32.5	Present	75.13	Positive	176	135	3	110	38	17	6	15	4	32	12	1
12	Low	<0.01	Absent	NA	NA	1884	285	655	366	136	20	11	6	10	32	2	0.4
13	Low	<0.01	Absent	NA	NA	2102	621	535	226	148	111	39	69	5	37	7	4
14	Low	0.3	Present	NA	NA	1013	126	418	291	188	147	16	158	4	34	4	12
15	High	14.3	Present	NA	NA	1066	159	513	195	120	79	59	48	59	21	2	0.4
16	High	5.4	Present	NA	NA	81	141	344	208	156	6	6	70	6	28	3	1
17	Low	0.3	Absent	NA	NA	1606	181	954	670	754	128	14	21	9	62	11	3
18	High	3.9	Absent	NA	NA	1755	201	219	198	298	91	39	320	106	146	NA	3
19	Low	0.01	Absent	NA	NA	1332	209	255	228	178	61	44	24	11	40	16	1
20	Low	0	Absent	NA	NA	2362	709	757	612	511	286	60	798	19	61	7	2
A	DNW	DNW	NA	55.17	NA	NA	NA	NA	NA	NA	NA	NA	NA	NA	NA	NA	NA
B	Low	<0.01	Absent	0.99	NA	NA	NA	NA	NA	NA	NA	NA	NA	NA	NA	NA	NA
C	High	71.6	Present	100.00	NA	NA	NA	NA	NA	NA	NA	NA	NA	NA	NA	NA	NA
D	High	29.4	Present	96.96	NA	NA	NA	NA	NA	NA	NA	NA	NA	NA	NA	NA	NA

*High viral cases showed >2% tissue area staining on RNA-ISH
** Interpreted as per supplementary table S7

Supplementary Table 5. Control cohort

Case number	Age(decade)	Gender	site/organ	Clinical comments - pulmonary disease	Pulmonary pathology	COVID- 19 RNA ISH	Total RNA sequence – SARS-CoV-2
1	50s	M	Lung	Hypoxemic respiratory failure likely secondary to worsening interstitial lung disease	Interstitial lung disease atherosclerotic coronary artery disease and ischemic heart disease.	negative	negative
2	50s	F	Lung	AML s/p SCT on immunosuppression, shock, respiratory distress	Organizing pneumonia	negative	negative
3	70s	F	Lung	Amyotrophic Lateral Sclerosis	Lungs with acute and chronic aspiration pneumonia	negative	negative
4	50s	F	Lung	NA	Diffuse alveolar damage	negative	negative
5	50s	M	Lung	Pneumonia, Ischemic cardiomyopathy	Pulmonary hemorrhage	negative	negative

Supplementary Table 6. Histopathological findings

Case number	# slides reviewed	hyaline membrane	alveolar cell hyperplasia	squamous metaplasia	interstitial organization	airspace organization	microvascular thrombosis	large vessel thrombosis	interstitial CI	vasculitis	acute pneumonia	DAD status
1	3	2	1	0	0	0	0	0	1	0	1	A
2	4	1	0	2	1	2	0	0	2	0	0	A+O
3	4	1	1	0	1	0	0	0	2	0	0	A+O
4												
5	5	1	1	2	1	0	1	0	1	0	0	A+O
6	5	1	1	1	1	0	0	0	1	0	2	A+O
7	5	1	0	0	0	0	0	0	1	0	2	A
8	5	0	0	0	0	0	1	0	1	0	0	N/A
9	4	2	1	0	1	0	0	0	1	0	0	A+O
10	5	0	0	0	0	0	2	0	1	0	0	N/A
11	5	2	0	0	0	0	0	0	1	0	1	A
12	5	1	0	0	1	1	0	0	1	0	0	A+O
13	5	1	0	0	0	0	0	0	1	0	0	A
14	5	2	1	2	2	2	2	0	1	0	0	A+O
15	4	1	0	0	0	0	0	0	1	0	1	A
16	5	2	1	0	1	0	1	0	1	0	0	A+O
17	5	1	1	1	1	1	0	0	2	0	2	A+O
18	5	0	0	0	0	0	0	0	0	0	0	N/A
19	5	0	0	0	2	2	0	0	1	0	0	O
20	5	0	0	1	0	1	0	0	1	1	0	O

0=absent, 1=mild, 2=marked DAD- diffuse alveolar damage, A- acute O- organizing, N/A = not available

Supplementary Table 7. Stranded RNA-seq read alignments for SARS-CoV-2 genes

Sample	Positive sense	Negative sense	% Positive sense	% Negative sense
Case1-lung1 LUL	478046	34114	93	7
Case1-lung2 RML	49673	4568	92	8
Case1-lung3 RUL	17972	2674	87	13
Case1-lung4 LLL	36140	880	98	2
Case1-heart1	0	0	0	0
Case2-lung1 RLL	408	60	87	13
Case2-lung2 LUL	42	0	100	0
Case2-lung3 RUL	10	0	100	0
Case2-heart1	0	0	0	0
Case2-jejunum1	10	0	100	0
Case3-lung1 LUL	10	4	71	29
Case3-lung2 RLL	4	0	100	0
Case3-heart1	0	0	0	0
Case3-liver1	0	0	0	0
Case4-lung1 LUL	0	0	0	0
Case4-lung2 RLL	0	12	0	100
Case4-heart1	0	0	0	0
Case4-heart2	0	0	0	0
Case4-liver1	0	0	0	0
Case4-bowel1	0	0	0	0
Case4-kidney1	0	0	0	0
Case5-lung1 LLL	218	0	100	0
Case5-lung2 RML	20	18	53	47
Case5-lung3 LUL	642	6	99	1
Case5-lung4 RML	512	2	100	0
Case5-lung5 RUL	680	32	96	4
Case5-heart1	0	0	0	0
Case5-liver1	6	0	100	0
Case5-bowel1	0	0	0	0
Case5-kidney1	0	0	0	0
Case5-marrow1	0	0	0	0
Case5-skin1	0	0	0	0
Case5-adipose1	0	0	0	0
Case6-lung1 LUL	14	0	100	0
Case6-lung2 LLL	0	0	0	0
Case6-lung3 RLL	0	0	0	0
Case6-lung4 RML	0	4	0	100
Case6-lung5 RUL	0	0	0	0
Case7-lung1 RLL	14	0	100	0
Case7-lung2 RML	146	8	95	5
Case7-lung3 LLL	24	2	92	8

Case7-lung4 RUL	0	0	0	0
Case7-lung5 LUL	1272	8	99	1
Case8-lung1 RLL	2990	156	95	5
Case8-lung2 RUL	98	102	49	51
Case8-lung3 LLL	23907	440	98	2
Case8-lung4 RML	5675	122	98	2
Case8-lung5 LUL	102	0	100	0
Case8- heart1	78	0	100	0
Case8- liver1	0	0	0	0
Case8- bowel1	24	0	100	0
Case9- lung1RLL	42337	2898	94	6
Case9- lung2 RML	138177	14600	90	10
Case9- lung3RUL	126143	11148	92	8
Case9-lung4 LUL	341214	17118	95	5
Case9- lung5LLL	152423	25962	85	15
Case10- lung1 LUL	26	0	100	0
Case10- lung2 LLL	112	0	100	0
Case10- lung3 RLL	22	0	100	0
Case10-kidney1	0	0	0	0
Case11-lung1RML	69278	2276	97	3
Case11-lung32RUL	14948	2178	87	13
Case11-lung3RLL	1282	46	97	3
Case11 -bowel1	92	0	100	0
Case11- kidney1	4	0	100	0
CaseA-lung	1144	48	96	4
CaseB-lung	4	2	67	33
CaseC-lung	165066	2534	98	2
CaseD-lung	40522	2982	93	7
Neg control1	0	0	0	0
Neg control2	0	0	0	0
Neg control3	0	0	0	0
Neg control4	0	0	0	0
Neg control5	0	0	0	0

Supplementary Table 8. Interpretation of qPCR

2019 nCoV_N1 probe	2019 nCoV_N2 probe	RNaseP	Interpretation	Report
+	+	±	2019-nCoV detected	Positive
If only one of the two targets is positive		±	Inconclusive Result	Inconclusive
-	-	+	2019-nCoV not detected	Negative
-	-	-	Invalid Result	Invalid

* positive result is defined by a CT value less then 40.00

Supplementary Table 9. Antibodies and epitope retrieval conditions for IHC staining

Protein Target	company	Clone	Antibody dilution	Antibody incubation	Epitope retrieval condition
CD3	Leica	LN10	Ready to use	15 minutes	ER2 for 15 minutes
CD8	Leica	4B11	Ready to use	15 minutes	ER2 for 20 minutes
CD20	Leica	L26	1:100	15 minutes	ER1 for 30 minutes
CD163	Leica	10D6	Ready to use	15 minutes	ER2 for 20 minutes
Napsin A	Leica	IP64	1:200	15 minutes	ER2 for 20 minutes
CD123	Leica	BR4MS	1:100	15 minutes	ER2 for 20 minutes
PDL1	Cell Signaling	E1L3N	1:200	15 minutes	ER2 for 20 minutes
IDO	Cell Signaling	D5J4E	1:400	30 minutes	ER2 for 20 minutes
SARS nucleocapsid	Novus Biologicals	NB100-56576	1:250	30 minutes	ER2 for 20 minutes
WS keratin	Dako	Z062201-2	1:900	15 minutes	ER2 for 20 minutes

Supplementary Table 10. Primer and Probes

Name	Description	Oligonucleotide Sequence (5'>3')	Label[1]	Final Conc.
2019-nCoV_N1-F	2019-nCoV_N1 Forward Primer	GAC CCC AAA ATC AGC GAA AT	None	500nM
2019-nCoV_N1-R	2019-nCoV_N1 Reverse Primer	TCT GGT TAC TGC CAG TTG AAT CTG	None	500nM
2019-nCoV_N1-P	2019-nCoV_N1 Probe	FAM-ACC CCG CAT TAC GTT TGG TGG ACC-BHQ1	FAM, BHQ-1	125nM
2019-nCoV_N1-P	2019-nCoV_N1 Probe	FAM-ACC CCG CAT /ZEN/ TAC GTT TGG TGG ACC-3IABkFQ	FAM, ZEN, 3IABkFQ	125nM
2019-nCoV_N2-F	2019-nCoV_N2 Forward Primer	TTA CAA ACA TTG GCC GCA AA	None	500nM
2019-nCoV_N2-R	2019-nCoV_N2 Reverse Primer	GCG CGA CAT TCC GAA GAA	None	500nM
2019-nCoV_N2-P	2019-nCoV_N2 Probe	FAM-ACA ATT TGC CCC CAG CGC TTC AG-BHQ1	FAM, BHQ-1	125nM
2019-nCoV_N2-P	2019-nCoV_N2 Probe	FAM-ACA ATT TGC /ZEN/ CCC CAG CGC TTC AG-3IABkF	FAM, ZEN, 3IABkFQ	125nM
RP-F	RNAse P Forward Primer	AGA TTT GGA CCT GCG AGC G	None	500nM
RP-R	RNAse P Reverse Primer	GAG CGG CTG TCT CCA CAA GT	None	500nM
RP-P	RNAse P Probe	FAM – TTC TGA CCT GAA GGC TCT GCG CG – BHQ-1	FAM, BHQ-1	125nM
RP-P	RNAse P Probe	FAM-TTC TGA CCT /ZEN/ GAA GGC TCT GCG CG-3IABkFQ	FAM, ZEN, 3IABkFQ	125nM

[1]TaqMan® probes are labeled at the 5′-end with the reporter molecule 6-carboxyfluorescein (FAM) and with the quencher, Black Hole Quencher 1 (BHQ-1) (Biosearch Technologies, Inc., Novato, CA) at the 3′-end. TaqMan® probes can also be labeled at the 5′-end with the reporter molecule 6- carboxyfluorescein (FAM) and with a double quencher, ZEN™ Internal Quencher positioned between the ninth (9th) and tenth (10th) nucleotide base in the oligonucleotide sequence and Iowa Black® FQ (3IABkFQ) located at the 3'-end (Integrated DNA Technologies, Coralville, IA).

Supplementary Table 11. Antibodies used for Immunofluorescent staining

Name	Channel	host	company	clone #	Catalog #	Concentration used
PanCK	488	mouse	Novus	AE1/AE3	NBP2-33200AF488	[1:500]
CD68	594	mouse	Santa Cruz	KP1	sc-20060AF594	[1:400]
CD45	647	mouse	Novus	2B11+PD7/26	NBP2-34528AF647	[1:200]
