# Supplementary material for: Temporal and spatial heterogeneity of host response to SARS-CoV-2 pulmonary infection
Source: Nat Commun. 2020 Dec 9;11:6319. doi: 10.1038/s41467-020-20139-7 (PMC7725958; doi:10.1038/s41467-020-20139-7)
Supplement: Supplementary file 3 — Descriptions of Additional Supplementary Files [file 41467_2020_20139_MOESM3_ESM.pdf]

Descriptions of Additional Supplementary Files

Supplementary Data 1

Description: Summary qRT PCR, RNA- ISH and RNA-Seq data

Supplementary Data 2

Description: Genes in each cluster for Figure 2

Supplementary Data 3

Description: Differential gene expression of high vs low virus cases
