# Supplementary material for: Temporal and spatial heterogeneity of host response to SARS-CoV-2 pulmonary infection
Source: Nat Commun. 2020 Dec 9;11:6319. doi: 10.1038/s41467-020-20139-7 (PMC7725958; doi:10.1038/s41467-020-20139-7)
Supplement: Supplementary file 7 — Reporting Summary [file 41467_2020_20139_MOESM7_ESM.pdf]

nature research

Corresponding author(s): James R. Stone, David T. Ting, Vikram Deshpande

Last updated by author(s): 10-21-2020

Reporting Summary

Nature Research wishes to improve the reproducibility of the work that we publish. This form provides structure for consistency and transparency in reporting. For further information on Nature Research policies, see our Editorial Policies and the Editorial Policy Checklist.

Statistics

For all statistical analyses, confirm that the following items are present in the figure legend, table legend, main text, or Methods section.

n/a	Confirmed	
☐	☒	The exact sample size (n) for each experimental group/condition, given as a discrete number and unit of measurement
☐	☒	A statement on whether measurements were taken from distinct samples or whether the same sample was measured repeatedly
☐	☒	The statistical test(s) used AND whether they are one- or two-sided<br>*Only common tests should be described solely by name; describe more complex techniques in the Methods section.*
☒	☐	A description of all covariates tested
☐	☒	A description of any assumptions or corrections, such as tests of normality and adjustment for multiple comparisons
☐	☒	A full description of the statistical parameters including central tendency (e.g. means) or other basic estimates (e.g. regression coefficient) AND variation (e.g. standard deviation) or associated estimates of uncertainty (e.g. confidence intervals)
☒	☐	For null hypothesis testing, the test statistic (e.g. F, t, r) with confidence intervals, effect sizes, degrees of freedom and P value noted<br>*Give P values as exact values whenever suitable.*
☒	☐	For Bayesian analysis, information on the choice of priors and Markov chain Monte Carlo settings
☐	☒	For hierarchical and complex designs, identification of the appropriate level for tests and full reporting of outcomes
☒	☐	Estimates of effect sizes (e.g. Cohen's d, Pearson's r), indicating how they were calculated

Our web collection on statistics for biologists contains articles on many of the points above.

Software and code

Policy information about availability of computer code

Data collection	No Software was used for data collection
Data analysis	FASTQC - 0.11.2, STAR - 2.6.1, Picard - 2.23.8, SAMtools - 1.9, HTSeq-count - 0.11.1, R - 3.0.2, DESeq2 - 1.28.1, CIBERSORTxV1.0.0, SpatialDecon R libraryV1.0.0

For manuscripts utilizing custom algorithms or software that are central to the research but not yet described in published literature, software must be made available to editors and reviewers. We strongly encourage code deposition in a community repository (e.g. GitHub). See the Nature Research guidelines for submitting code & software for further information.

Data

Policy information about availability of data

All manuscripts must include a data availability statement. This statement should provide the following information, where applicable:

- Accession codes, unique identifiers, or web links for publicly available datasets
- A list of figures that have associated raw data
- A description of any restrictions on data availability

Sequencing datasets generated during and/or analysed during the current study are available in the GEO repository, https://www.ncbi.nlm.nih.gov/geo/query/acc.cgi?acc=GSE150316. NanoString GeoMX data is available in GEO repository , accession number GSE159788

Field-specific reporting

Please select the one below that is the best fit for your research. If you are not sure, read the appropriate sections before making your selection.

☒ Life sciences ☐ Behavioural & social sciences ☐ Ecological, evolutionary & environmental sciences

For a reference copy of the document with all sections, see nature.com/documents/nr-reporting-summary-flat.pdf

Life sciences study design

All studies must disclose on these points even when the disclosure is negative.

Sample size	No sample size calculation was performed. This study was performed during the COVID-19 pandemic on subsequent 20 autopsy specimens at MGH and 4 from Columbia, NYC from patients who succumbed to SARS-CoV-2 infection. Given the nature of SARS-CoV-2, autopsy was performed under special infection control protocols. The intent of the study was discovery and the uncertainty of patients going to autopsy unpredictable. Therefore, no prespecified number of patients was selected.
Data exclusions	no data exclusions apply
Replication	Each experiment was performed once.
Randomization	This study is on autopsy material from patients who succumbed to SARS-CoV-2 infection to look for pathogenesis and other finding. No treatment or intervention was performed excluding need for randomization.
Blinding	This study is on autopsy material from patients who succumbed to SARS-CoV-2 infection to look for pathogenesis and other findings. No treatment or intervention was performed excluding need for blinding.

Reporting for specific materials, systems and methods

We require information from authors about some types of materials, experimental systems and methods used in many studies. Here, indicate whether each material, system or method listed is relevant to your study. If you are not sure if a list item applies to your research, read the appropriate section before selecting a response.

Materials & experimental systems

n/a	Involved in the study	
☐	☒	Antibodies
☒	☐	Eukaryotic cell lines
☒	☐	Palaeontology and archaeology
☒	☐	Animals and other organisms
☐	☒	Human research participants
☒	☐	Clinical data
☒	☐	Dual use research of concern

Methods

n/a	Involved in the study	
☒	☐	ChIP-seq
☒	☐	Flow cytometry
☒	☐	MRI-based neuroimaging

Antibodies

Antibodies used	CD3-Leica,LN10. CD8-Leica, 4B11. CD20-Leica, L26. CD163-Leica, 10D6. Napsin A- Leica, IP64. CD123-Leica, BR4MS. PDLl-Cell Signaling, Ell3N. IDO- Cell Signaling, D5J4E. SARS nucleocapsid- Novus Biologicals, NBl00-56576. WS keratin- Dako, 2062201-2, PanCK-Novus, CD68-Santa Cruz, CD45-Novus.
Validation	IDOl- Single-Cell Atlas Reveals Complexity of the lmmunosuppressive Microenvironment of Initial and Recurrent Glioblastoma. PMID: 32457755 PMCID: PMC7221162 DOI: 10.3389/fimmu.2020.00835<br>SARS nucleocapsid- Pathology and Pathogenesis of SARS-CoV-2 Associated with Fatal Coronavirus Disease, United States. PMID: 32437316 DOI: 10.3201/eid2609.202095<br>CD3 -. National Committee for Clinical Laboratory Standards (NCCLS). Protection of laboratory workers from infectious diseases transmitted by blood and tissue; proposed guideline. Villanova, P.A. 1991; 7(9). Order code M29-P.<br>CD8- Williamson SLH, Steward M, Milton I, et al. New monoclonal antibodies to the T cell antigens CD4 and CD8. American Journal of Pathology 1998;152(6):1421-1426.<br>CD20-Chen CC, Raikow RB, Sonmez-Alpan E et al. Classification of small B-cell lymphoid neoplasms using a paraffin section immunohistochemical panel. Applied Immunohistochemistry Molecular Morphology. 2000; 8(1):1–11.<br>CD163- Jung KY, Cho SW, Kim YA, et al. Cancers with higher density of tumor-associated macrophages were associated with poor survival rates. Journal of Pathology and Translational Medicine. 2015; 49(4): 318-324.<br>Napsin A- Kandalaft P.L, Gown A.M, Isacson C. The lung-restricted marker napsin A is highly expressed in clear cell carcinomas of the ovary. American Journal of Clinical Pathology. 2014; 142: 830-836.<br>CD123- Garnache–Ottou F, Feuillard J and Saas P. Plasmacytoid dendritic cell leukaemia/lymphoma: towards a well defined entity?

British Journal of Haematology. 2007; 136:539–548.
PD:-1 - Stephen T Ryan, et. al. Neoadjuvant rituximab modulates the tumor immune environment in patients with high risk prostate cancer. J Transl Med. 2020
WS Keratin - Pinkus GS, et al. Optimal immunoreactivity of keratin proteins in formalin-fixed, paraffin-embedded tissue requires preliminary trypsinization: An immunoperoxidase study of various tumors using polyclonal and monoclonal antibodies. J Histochem Cytochem 1985; 33:465

Human research participants

Policy information about studies involving human research participants

Population characteristics	The mean age of this cohort was 62.5 years (range 32-89) with 14 males and 10 females. All 24 cases were confirmed for SARS-CoV-2 infection through qRT-PCR assays performed on nasopharyngeal swab specimens as part of clinical care.
Recruitment	Patients were not recruited, but this was a discarded tissue protocol from autopsy. Given the patients are deceased, they are no longer considered human subjects. As stated, these were sequential autopsies at the MGH and IRB review was obtained for discarded tissue analysis.
Ethics oversight	Analysis of patient autopsy material was reviewed and approved by the Partners Human Research IRB (Protocol#: 2020P001001).

Note that full information on the approval of the study protocol must also be provided in the manuscript.
